# Evaluation of Asymptomatic Peripheral Arterial Disease by Ankle- Brachial Index in Patients with Concomitant Coronary Arterial Disease

**Published:** 2012-12-15

**Authors:** Hosein Vakili, Roxana Sadeghi, Kobra Doustali, Habibollah Saadat, Mohammad Hasan Namazi, Morteza Safi

**Affiliations:** 1 Cardiovascular Research Center, Shahid Beheshti University of Medical Sciences, Tehran, IR Iran; 2 Department of Cardiovascular , Loghman Hakim Hospital, Shahid Beheshti University of Medical Sciences, Tehran, IR Iran

**Keywords:** Ankle-Brachial Index, Peripheral Arterial Disease, Coronary Artery, Asymptomatic

## Abstract

**Background:**

Peripheral arterial disease is associated with adverse cardiovascular outcomes. As such, it is found that screening for peripheral arterial disease (PAD) improves risk assessment. Thus, intensive risk factor modification and medical treatment in these patients are necessary.

**Objectives:**

The purpose of this study was to determine the prevalence of asymptomatic peripheral arterial disease in patients with concomitant coronary arterial disease.

**Methods:**

Asymptomatic peripheral arterial disease was investigated in 400 patients (60% males, 40% females, aged 59.7± 11.3) with a documented coronary arterial disease.

**Results:**

Among patients with documented CAD, 12% had asymptomatic PAD with the ABI ratio of less than 0.9.

**Conclusions:**

It is advisable to screen for PAD not only as a disease but also as a risk assessment method for atherosclerosis.

## 1. Introduction

Peripheral arterial disease (PAD) is narrowing of lower-limb arteries which leads to compromised blood circulation in affected areas.

The presence of PAD among patients with coronary arterial disease (CAD) is associated with more progressive atherosclerosis and adverse cardiovascular outcomes (1,2).

Indeed, the majority of patients with PAD remains asymptomatic and under diagnosed. They can be identified by inexpensive and non-invasive techniques. This study emphasized the need for PAD screening and intensive risk factor modification in patients with PAD.

The objective of the current study was to determine the prevalence of asymptomatic and under diagnosed PAD in patients with documented CAD.

## 2. Materials and Methods

### 2.1. Study population

This was a cross-sectional descriptive-analytical study and comprised 400 CAD patients with no symptoms of PAD. All patients were admitted to our hospital for elective coronary angiography. PAD was determined on the basis of a low ankle-brachial index (ABI), lower than <0.9. The study was approved by the Ethics Committee of Cardiovascular Research Center of Shahid Beheshti University of Medical Sciences and informed consent was obtained from all patients before enrollment.

### 2.2. ABI

The ABI measurement was performed after the patient has been rested in supine position for 10 minutes.

However, different methods are reported for the calculation of ABI (3). In this study as suggested by the American Heart Association (AHA) (4), the ABI was determined by dividing the higher of the two systolic blood pressures at the ankle by the higher of the two brachial artery systolic pressures as described below:

ABI=Higher ankle systolic blood pressure / Higher brachial systolic blood pressure

### 2.3. Statistical analysis

Patients with documented CAD were stratified by the presence or absence of PAD. Results were reported as percentages for categorical variables and mean ± SD for continuous variables. Demographic and clinical characteristics were compared by using Pearson’s Chi-square test for categorical variables and student t test or analysis of variance for continuous variables as appropriate. A two-sided P<0.05 was considered significant.

## 3. Results

Patients’ characteristics: This study comprised 400 patients with documented CAD including 240 men (60%) and 160 women (40%) aged from 30 to 80 years, with mean age of 59.7± 11.3. PAD was found in 48 (12%) patients with CAD. The clinical characteristics of CAD patients with or without PAD were depicted in table 1. There was no significant difference between groups in regard to gender. The CAD patients with PAD were older (67.56± 11.37 years vs. 58.65± 10.88 years, P< 0.001), and more likely to have diabetes (52.1 % vs. 26.6 %, P< 0.001, OR= 3.5), and hypertension (68.7% vs. 49.1%, P< 0.02, OR= 2.3). There was also no difference between groups in terms of hyperlipidemia (45.9 % vs. 40.3 %, P= 0.283) and smoking history (27.1% vs. 34.4 %, P= 0.5).

**Table 1:** Clinical Characteristics of CAD Patients with or without PAD

Parameter	CAD with PAD (n = 48, 12%)	CAD without PAD (n = 352, 88%)	P Value
Age, yrs	58.65 ± 10.88	67.56 ± 11.37	< 0.001
Sex, female	21, 43.8	139, 39.5	= 0.33
Diabetes mellitus	25, 52.1	83, 26.6	< 0.001
Hypertension	33, 68.7	173, 49.1	< 0.02
Hyperlipidemia	22, 45.9	142, 40.3	= 0.283
Smoking	13, 27.1	121, 34.4	= 0.5
3VD ^[Table-fn fn5652]^	39, 81.3	103, 29.3	< 0.001
CABG	11, 23	24, 6.8	< 0.001

Abbreviations: 3VD, three vessel disease in coronary angiography; CABG, Coronary Artery Bypass Graft

Additionally, PAD patients tended to have angiographic three-vessel coronary disease (81.3 % vs. 29.3 %, P< 0.001, OR= 10.4) and were more likely to have a prior CABG (23% vs. 6.8%, P< 0.001, OR= 4).

## 4. Discussion

Previous studies have demonstrated clinical importance of PAD, which has similar frequency in men and women and increases with age (5-7). Risk factors for PAD are similar to other atherosclerotic diseases. Patients with PAD have more extensive atherosclerosis, and experience greater progressive disease. These findings continue even after matching for cardiovascular risk factors (8,9).

The presence of PAD in patients with concomitant CAD shows an aggressive form of atherosclerosis. Thus, detection of PAD in the CAD patients emphasizes the need for more aggressive risk factor modification.

General awareness of PAD is lower than any other atherosclerotic diseases such as stroke, coronary artery disease or heart failure and PAD is frequently under diagnosed(10). Epidemiological studies have found that asymptomatic PAD is three to four times more common than symptomatic types(11,12). In asymptomatic PAD, more patients have been missed or received suboptimal treatment. In multiple studies, the presence of intermittent claudication underestimated the percent of PAD(13-16).

Consequently, ABI is a simple and inexpensive test for evaluation of the PAD. It measures the ratio of blood pressure in the lower relative to that of the upper limbs.

ABI is not only a simple technique for PAD screening, but it also has a good inter-observer reliability(17). Normal ABI values generally range from 0.9-1.3. The sensitivity and specificity of an ABI<0.9 for detection of ≥ 50% stenosis in the peripheral arteries are 90% and 98%, respectively(18). The prognostic value of ABI is similar to the Framingham risk factors for cardiovascular morbidity and mortality(19). ABI less than 0.9 is an independent risk factor for cardiovascular mortality across all Framingham risk factors(20).

Post-exercise measurement of ABI might be recommended in addition to the resting ABI evaluation. Exercise may unmask some patients with mild PAD(21).

Some limitations of ABI include failure to detect the PAD in patients with severe aortoiliac stenosis or occlusion, and a rich collateral network. Furthermore, for patients with severe arterial wall calcification, the arteries were incompressible, and thus ABI could not diagnose the PAD. For this group of patients, toe brachial index might be helpful since toe arteries are rarely calcified.

## Conclusion

In our study, 12% of patients with documented CAD had asymptomatic PAD. The presence of PAD is associated with adverse cardiovascular outcomes. So, it is reasonable to use the ABI not only as a diagnostic method but also as a risk assessment tool. Improving PAD detection is crucial for more effective cardiovascular prevention and treatment.
